# Chronic, intermittent treatment with a cannabinoid receptor agonist impairs recognition memory and brain network functional connectivity

**DOI:** 10.1111/jnc.14549

**Published:** 2018-09-27

**Authors:** Francisco M. Mouro, Joaquim A. Ribeiro, Ana M. Sebastião, Neil Dawson

**Affiliations:** ^1^ Faculdade de Medicina Instituto de Farmacologia e Neurociências Universidade de Lisboa Lisboa Portugal; ^2^ Faculdade de Medicina Instituto de Medicina Molecular Universidade de Lisboa Lisboa Portugal; ^3^ Division of Biomedical and Life Sciences University of Lancaster Lancashire UK

**Keywords:** cannabinoids, chronic, functional connectivity, recognition memory

## Abstract

Elucidating how cannabinoids affect brain function is instrumental for the development of therapeutic tools aiming to mitigate ‘on target’ side effects of cannabinoid‐based therapies. A single treatment with the cannabinoid receptor agonist, WIN 55,212‐2, disrupts recognition memory in mice. Here, we evaluate how prolonged, intermittent (30 days) exposure to WIN 55,212‐2 (1 mg/kg) alters recognition memory and impacts on brain metabolism and functional connectivity. We show that chronic, intermittent treatment with WIN 55,212‐2 disrupts recognition memory (Novel Object Recognition Test) without affecting locomotion and anxiety‐like behaviour (Open Field and Elevated Plus Maze). Through ^14^C‐2‐deoxyglucose functional brain imaging we show that chronic, intermittent WIN 55,212‐2 exposure induces hypometabolism in the hippocampal dorsal subiculum and in the mediodorsal nucleus of the thalamus, two brain regions directly involved in recognition memory. In addition, WIN 55,212‐2 exposure induces hypometabolism in the habenula with a contrasting hypermetabolism in the globus pallidus. Through the application of the Partial Least Squares Regression (PLSR) algorithm to the brain imaging data, we observed that prolonged WIN 55,212‐2 administration alters functional connectivity in brain networks that underlie recognition memory, including that between the hippocampus and prefrontal cortex, the thalamus and prefrontal cortex, and between the hippocampus and the perirhinal cortex. In addition, our results support disturbed lateral habenula and serotonin system functional connectivity following WIN 55,212‐2 exposure. Overall, this study provides new insight into the functional mechanisms underlying the impact of chronic cannabinoid exposure on memory and highlights the serotonin system as a particularly vulnerable target.

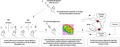

**Abbreviations used**
^14^C‐2‐DG: ^14^C‐2‐deoxyglucose
AcbC: nucleus accumbens core
AcbSh: nucleus accumbens shell
AM: anteromedial thalamus
aPrL: anterior prelimbic cortex
aRT: reticular thalamus
AV: anteroventral thalamus
BLA: basolateral amygdala
C57BL/6: black‐six mice
CB_1_R: cannabinoid receptor 1
Cg1: cingulate cortex
ChVeh: chronic VEH
ChWin: chronic WIN 55,212‐2
CoA: central Amygdala
DH CA1: dorsal Hippocampus, CA1
DH CA2: dorsal Hippocampus, CA2
DH DG: dorsal Hippocampus, Dentate Gyrus
DH Mol: dorsal Hippocampus, Molecular Layer
DLO: dorsolateral orbital cortex
DLST: dorsolateral striatum
DMSO: dimethylsulfoxide
DR: dorsal raphe
DSub: dorsal subiculum
ENT: entorhinal cortex
EPMT: elevated plus maze test
fMRI: functional magnetic resonance imaging
FRA: frontal association area
GP: globus pallidus
Hab: habenula
HDB: horizontal limb of diagonal band of Broca
I.P: intraperitoneal injection
IL: infralimbic cortex
Ins: insular cortex
LCGU: local cerebral glucose utilization
LO: lateral orbital cortex
LS: lateral septum
MB: mammillary body
MD: medio‐dorsal thalamus
MeA: medial amygdala
MG: medial geniculate
MO: medial orbital cortex
mPrL: medial prelimbic cortex
MR: medial raphe
MS: medial septum
MTL: medial temporal lobe
NORT: novel object recognition test
NPI: novelty preference index
OFT: open field test
PD: post‐natal days
Piri: piriform cortex
PLSR: partial least squares regression
PRh: perirhinal cortex
RSc: retrosplenial cortex
S1: somatosensory cortex
SNC: substancia nigra pars compacta
SNR: substancia nigra pars reticulata
UR: uptake ratio
VDB: ventral limb of diagonal band of broca
VH CA1: ventral hippocampus, CA1
VH CA3: ventral hippocampus, CA3
VH DG: ventral hippocampus, dentate gyrus
VH LMol: ventral hippocampus, molecular layer
VL: ventrolateral thalamus
VMST: ventrolateral striatum
VM: ventromedial thalamus
VTA: ventral tegmental area
WIN55,212‐2: (*R*)‐(+)‐[2,3‐dihydro‐5‐methyl‐3‐(4‐morpholinylmethyl)pyrrolo[1,2,3‐*de*]‐1,4‐benzoxazin‐6‐yl]‐1‐naphthyl‐methanone mesylate

Heavy or regular cannabis abuse, generally defined as daily or almost‐daily use over a prolonged period of time, has been linked to cognitive dysfunction (Abush and Akirav 2012) and increased risk of developing psychiatric symptoms, including schizophrenia spectrum disorders (Andréasson *et al*. 1987; Hall and Degenhardt 2009), acute psychosis and mania (Khan and Akella 2009) and an amotivational syndrome (Tunving 1987; Fujiwara 2001; Ozaki and Wada 2001). In addition, cannabis‐based medicines are increasingly being used to treat several diseases such as epilepsy (Maa and Figi 2014), chronic pain (Carter *et al*., 2015), multiple sclerosis (Fitzpatrick and Downer 2017) and neurodegenerative diseases (Fagan and Campbell 2014), but the potential for negative side effects has not been well characterized. Understanding the effects of chronic cannabinoid exposure upon brain and synaptic function opens a window into the development of therapeutic tools that could counteract the ‘on target’ side effects associated with chronic use of cannabis and cannabinoid‐based medicines (Copeland *et al*. 2013; Lovelace *et al*. 2015).

Cannabinoid receptor 1 (CB_1_R) mediates the characteristic psychoactive effects of exogenous cannabinoids and the synaptic actions of endocannabinoids (Kano *et al*. 2009). One immediate consequence of cannabis consumption is an impairment in memory consolidation, seen in both humans (Ranganathan and D'Souza 2006; Borgelt *et al*. 2013) and laboratory animals (Clarke *et al*. 2008; Kano *et al*. 2009; Sousa *et al*. 2011; Mouro *et al*. 2017). Cannabinoid‐mediated disruptions in learning and memory may be related to reported impairments in long‐term potentiation at glutamatergic synapses (Terranova *et al*. 1995; Stella *et al*. 1997; Misner and Sullivan 1999; Wang *et al*. 2016; Silva‐Cruz *et al*. 2017), detrimental modifications in fast‐/slow‐wave oscillations, known to be modulated by GABAergic interneurons (Freund *et al*. 2003), and altered activity in septal‐hippocampal monoaminergic and cholinergic pathways, known to regulate cortical plasticity and activity (Miller and Branconnier 1983; Gessa *et al*. 1998; Sullivan 2000; Redmer *et al*. 2003; Khakpai *et al*. 2012).

Studies in humans, using functional magnetic resonance imaging, have shown that chronic cannabis users display significant alterations in functional connectivity in brain networks relevant to self‐awareness (Pujol *et al*. 2014), working memory (Cousijn *et al*. 2013) and recognition memory (Riba *et al*. 2015) which may be linked with functional differences in structures of the medial temporal lobe and prefrontal cortex (PFC) (Riba *et al*. 2015). In a recent study, it was also shown that chronic marijuana use leads to increased functional connectivity in the orbitofrontal network, as well as higher functional connectivity in tracts that innervate the orbitofrontal cortex (Filbey *et al*. 2014). However, chronic consumption studies in humans can be contaminated by confounding variables, such as lifestyle factors or mixed drug use.

This work was designed to elucidate the impact of chronic, intermittent cannabinoid exposure on brain metabolism, functional brain connectivity and recognition memory. We carry out three different analysis: first, we evaluated recognition memory in the Novel Object Recognition Test (NORT); secondly, we studied brain metabolic activity in these animals using ^14^‐C‐2‐deoxyglucose (^14^C‐2‐DG) functional brain imaging and finally, we characterized alterations in brain network functional connectivity through the application of the Partial Least Squares Regression (PLSR) algorithm to the ^14^C‐2‐DG brain imaging data. We found out that adult mice chronically exposed to WIN 55,212‐2 displayed impaired recognition memory and differences in metabolic brain activity and dysfunctional connectivity in circuits that underlie memory processing, thus providing new insights into the functional mechanisms that underlie the impact of chronic cannabinoid exposure on memory.

## Methods

### Animals

Adult (8–12 weeks old) male C57BL/6 mice (IMSR Cat# CRL:27, RRID:IMSR_CRL:27) (Charles River, Barcelona, Spain) were used. Animals were housed in a temperature (22/24°C) and humidity (45–65%) regulated room with a 14/10‐h light/dark cycle (07:00–21:00) with *ad libitum* access to food and water. Animal behaviour experiments were conducted during the light phase at around the same time each day (10 : 00). All experimentation followed the European Community Guidelines (Directive 2010/63/EU) and the Portuguese law (DL 113/2013) for Animal Care for Research Purposes, and were approved by the ‘Instituto de Medicina Molecular’ Internal Committee and the Portuguese Animal Ethics Committee – Direcção Geral de Veterinária. The experimental protocol was not preregistered. Animals were habituated to the presence of the investigator and handled for 5 days before testing. A total of 40 mice were used, 20 were treated with WIN 55,212‐2 and 20 were receiving the vehicle only. All animals were used for behaviour analysis, which was performed in two series of 10 mice per drug condition, giving a total of 20 mice per condition. For connectivity analysis, 10 mice per condition were used. To determine whether an animal would be allocated to the control or experimental treatment group a pseudo‐randomization procedure was employed. Animals were tagged and distributed in groups of five animals to each housing cage (8 cages of 5 animals). Subsequently, four cages were randomly attributed to each treatment condition (either control or chronic WIN 55,212‐2). To do so, a number was attributed to each cage and randomly drawn. Drawn cage numbers were distributed sequentially to either the control or the experimental group (first drawn number allocated to control, the second drawn number attributed to experimental group, until all numbers have been drawn).

### Drugs

WIN 55,212‐2 (TOCRIS Bioscience, catalogue number 1038, Bristol, Avon, United Kingdom) was suspended in dimethylsulfoxide (Sigma Aldrich, catalogue number D2650, Missouri, St. Louis, MO, USA at stock concentration of 100 mM, and stored at −20°C; appropriate dilutions of these solutions were made in saline (NaCl 0.9%) before injection. The amount of dimethylsulfoxide present in the solutions used for intraperitoneal (*i.p*.) injections was < 0.6 μL per mouse, and control animals were injected with equivalent amounts of vehicle (2 mL/kg).

### Chronic WIN 55,212‐2 treatment protocol

**Figure 1:**
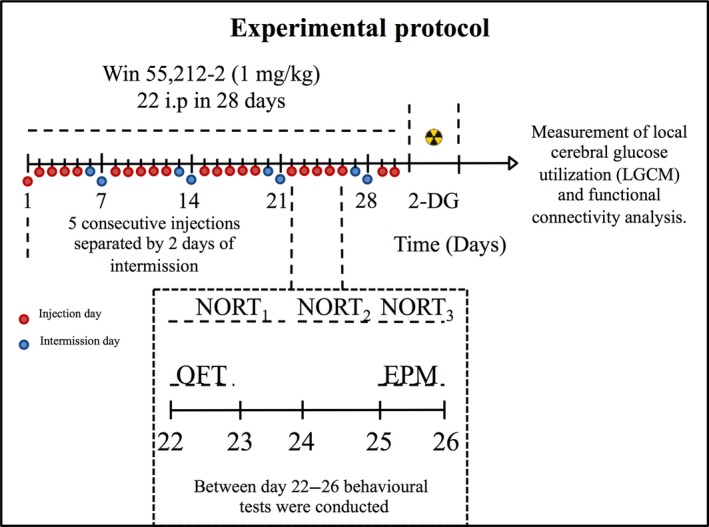
Scheme depicting the treatment schedule and overall experimental protocol. Over 30 consecutive days mice were treated with 22 intraperitoneal (i.p.) injections with WIN 55,212‐2 or vehicle control. Behavioural tests were performed between days 22 and 26; as indicated in the inset. The habituation phase of novel object recognition test (NORT) was initiated on day 22, simultaneously with the open field test (OFT). The habituation continued on the days 23 and 24. The training phase of NORT was performed on day 25. On day 26 the test phase of NORT was performed. Immediately after the NORT animals were placed in the EPM to assess anxiety‐like behaviour. Subsequently, 24 h after the final WIN 55,212‐2 treatment, animals underwent the ^14^C‐2‐deoxyglucose brain imaging protocol. From the initial 40 animals, one animal of the control group was excluded by post‐analysis (outlier, *p* < 0.05); thus, 19 control and 20 WIN 55,212‐2‐treated mice were used in the behavioural assays and 10 control and 10 WIN 55,212‐2‐treated mice were used in the ^14^C‐2‐deoxyglucose brain imaging study.

WIN 55,212‐2 was administered at a dose of 1 mg/kg *i.p*., a dose known to affect recognition memory in the NORT without creating sedative or cataleptic effects that are associated with higher doses (Schneider and Koch 2003; Baek *et al*. 2009; Mouro *et al*. 2017), over 30 days. Behavioural tests were carried out on the last 7 days of treatment. The injections were performed around the same hour of the day (18 : 00 ± 1 h), while behavioural tests were performed during the morning (10 : 00). Doing so, we avoided withdrawal symptoms (Maldonado 2002; Lichtman and Martin 2005; Solymosi and Köfalvi 2017), while minimizing acute effects of the drug during testing. In order to replicate a pattern of chronic intermittent cannabinoid exposure (Lamarque *et al*. 2001; Schneider and Koch 2003) animals were treated for 5 consecutive days followed by 2 days without treatment (22 injections over 30 days). This protocol was designed to minimize the tolerance to WIN 55,212‐2 that may develop during chronic continuous administration (see Maldonado 2002; Hampson *et al*. 2003; Solymosi and Köfalvi 2017). For further detail on the treatment and experimental protocol see Fig. 1.

### Novel object recognition test (NORT)

NORT was conducted in a wooden square open field arena (40 × 40 × 40 cm) as previously reported (Bailey and Crawley 2009; Antunes and Biala 2012). In brief, the test involved a habituation period (3 days), a training day and a test day. Before training, animals were habituated to the arena in the absence of any stimulus or object, under the same lighting and environmental conditions, for 20 min over 3 consecutive days. On the fourth day, after habituation, animals were placed inside the arena, facing away from the two identical objects (familiar objects) and allowed to freely explore the environment and objects for 5 min. After a retention interval of 24 h, to test long‐term memory (Clarke *et al*. 2008; Antunes and Biala 2012), animals were placed inside the arena with one novel and one familiar object (test day). Animals were allowed to explore the objects for 5 min. The objects used in the training and test days were wooden dolls (7 cm height × 6 cm width). The role of the object, as either familiar or novel, was randomized as was the location of their presentation. To randomize the role of the object a blind experimenter was asked to randomly select one of the objects to be the novel object for the first animal. Then, for the following animals, the novel object was always permuted, to ensure that both objects were used as the novel object the same number of times in both the control and the experimental groups (for instance, if the object A was randomly selected for the trial of animal 1, then for animal 2 the novel object would be object B, for animal 3 the object would be again object A, and so on). Between every trial the arena and the objects were cleaned with 30% ethanol to erase any olfactory clues. The objects were secured to the bottom of the arena with Velcro, which could not be seen or touched by the animals, and were placed in opposite corners of the arena. Activity was recorded using the video‐tracking software – SMART^®^ (RRID:SCR_002852). To refine the results obtained by software measures, a post‐analysis was conducted. In the post‐analysis, the investigator was blind to the experimental condition. Exploratory behaviour was quantified as the amount of time (seconds) animals spent investigating each object (only direct approaches were considered; ≤ 1 cm distance). The number of approaches that included sniffing the object, rearing towards the object or touching the object was counted (Ennaceur and Delacour 1988; Ennaceur 2010; Antunes and Biala 2012). Exploration of each object was quantified as the novelty preference index (NPI) – calculated as (B−A)/(B+A), where B corresponds to the time spent exploring the novel object and A the time spent exploring the familiar object, during the test phase of NORT. This index ranges from −1 to 1 (−1 = exclusive exploration of the familiar object; 0 = absence of discrimination between novel and familiar objects, i.e. equal time exploring both objects; and 1 = exploration of the novel object only). We defined full immobility during the test stage of NORT as an *a priori* exclusion criterion, by which no animals were excluded from analysis. However, data from one animal on the control group were eliminated by post‐analysis as it corresponded to a significant outlier (*p* < 0.05).

### Open field test (OFT) and elevated plus maze (EPM)

Anxiety‐like and locomotor behaviour were analysed in the OFT and EPM. Behaviour in the OFT was analysed before the NORT. The OFT was used to assess individual behaviour when animals were placed in a novel environment (Wilson *et al*. 1976), as well as anxiety (Careau *et al*. 2012). As locomotor activity can impact exploratory drive (Broadhurst 1957, 1958; Stanford 2007), it was important to ascertain that animals did not display significant differences in locomotor activity. The OFT took place in the same arena as that used for the NORT (square open field arena: 40 × 40 × 40 cm) and activity was recorded during the first time that animals had contact with the environment, that is, on the first 5 min of the first day of NOR habituation phase (see schematics on Fig. 1). To quantify behaviour, the percentage of time spent in the central zone of the arena was used as an indicator of anxiety, as previously described (Mouro *et al*. 2017). Mean velocity and distance moved were quantified to compare locomotor abilities between the experimental groups. Activity was recorded and analysed using the video‐tracking software – SMART^®^ (RRID:SCR_002852). The reference point used by the software to determine the position of the animal was the centre of the mouse dorsum, as done previously in our Institute (Batalha *et al*. 2013; Coelho *et al*. 2014; Mouro *et al*. 2017 ). Environmental conditions and animal manipulation procedures were kept as similar as possible between animals.

Anxiety‐like behaviour was also assessed in the EPM. Immediately after being tested in the NORT during the test day (see schematics on Fig. 1), animals were placed in a maze shaped like a plus sign composed by two open arms with no walls (5 × 29 cm) and two closed arms (5 × 29 × 15 cm) arranged perpendicularly and elevated 50 cm above the floor (Mouro *et al*. 2017). Animals were placed on the centre of the maze, facing an open arm, and allowed to freely explore the maze during 5 min. The total time spent in open arms was used as a measure of anxiety‐like behaviour (Pellow *et al*. 1985; Coelho *et al*. 2014; Mouro *et al*. 2017).

### ^14^C‐2‐D‐deoxyglucose functional brain imaging

Local cerebral glucose utilization (LCGU) was determined after the final WIN 55,212‐2 (or vehicle) treatment (*n* = 10 per group, at day 31 for half of the controls and half of the test animals, at day 32 for the remaining animals), in accordance with previously published protocols (Dawson *et al*. 2012, 2015). In brief, mice were injected *i.p*. with 4.625 MBq/Kg of 2‐deoxy‐D‐[^14^C]glucose diluted in physiological saline (American Radiolabeled Chemicals Inc., USA, catalogue number ARC 0111A, dose volume of 2.5 mL/kg). After the injection animals were returned to their home cages. Forty‐five minutes after isotope injection, animals were killed by cervical dislocation and subsequently decapitated. A terminal blood sample was collected from the neck by torso inversion, to determine circulating glucose levels (Accu‐Chek Aviva Blood Glucose Monitor). The brain was dissected out and immediately frozen in isopentane (−40°C) and stored at −80°C until sectioning. Blood samples were centrifuged to separate the plasma for determination of plasma 2‐deoxy‐D‐[^14^C]‐glucose concentration by liquid scintillation analysis (Table S1).

Brains were coronally sectioned in a cryostat (−20°C) at 20 μm. Three consecutive sections were retained from every 60 μm and dried rapidly onto slide covers on a hot plate (70°C). Autoradiograms were obtained by placing these sections, along with pre‐calibrated ^14^C‐standards (39‐1069 nCi/g tissue equivalents; American Radiolabelled Chemicals Inc., St. Louis, MO, USA, catalogue number ARC 0146R) to high‐resolution autoradiographic film (Carestream Kodak Biomax MR, Sigma‐Aldrich, UK, catalogue number Z358460‐50EA) for 7 days, after which they were developed in accordance with the manufacturer's instructions. Autoradiographic images were analysed by computer‐based images analysis (MCID/M5) (RRID:SCR_014278). The local isotope concentration for each brain region of interest (RoI) was obtained directly from the optical density of autoradiographic images relative to that of co‐exposed ^14^C‐standards. Forty‐nine anatomically distinct RoI were measured with reference to a stereotactic mouse brain atlas (Paxinos and Franklin 2001) (RRID:SCR_007127). LCGU in each RoI was obtained by comparing the ratio of ^14^C in each RoI to the ^14^C concentration in the whole brain of the same animal, referred to as the ^14^C‐2‐DG uptake ratio (^14^C‐2‐DG UR). The whole brain average ^14^C concentration was determined as the average ^14^C concentration across all sections in which a RoI was measured. No animals were excluded from this data set.

### Functional brain connectivity

Regional functional connectivity was analysed in control (saline treated) and WIN 55,212‐2‐treated mice. To elucidate the impact of chronic WIN 55,212‐2 exposure on regional functional connectivity, we applied the Partial Least Squares Regression (PLSR) algorithm (pls package in R, https://CRAN.R-project.org/package=pls, Mevik and Wehrens 2007) to characterize statistically significant differences in the functional connectivity of defined ‘seed regions’ to all the other RoI's analysed. In our analysis these seed regions were chosen on the basis of the overt differences in LCGU seen following chronic WIN 55,212‐2 treatment (Fig. 4). Thus, the chosen seed brain regions in our analysis were the dorsal subiculum of the ventral hippocampus (DSub), the mediodorsal thalamic nucleus (MD), the habenula (Hab) and the globus pallidus (GP). The application of the PLSR algorithm to functional ^14^C‐2‐DG imaging data was undertaken as previously reported (Dawson *et al*. 2012, 2015). In brief, functional connectivity between each seed RoI and all other RoI measured was defined by the variable importance to the projection (VIP) statistic gained from PLSR analysis. A significant functional connection between brain regions was considered to exist if the 95% confidence interval (CI) of the VIP statistic exceeded 0.8, denoting a considerable contribution of the explanatory variable (RoI) to the dependent variable (seed region) in PLSR analysis. The SD and CI of the VIP statistic were estimated by jack‐knifing. The significance of WIN 55,212‐2‐induced alterations in the VIP statistic was determined by *t*‐test with *post hoc* Bonferroni correction for multiple comparisons. WIN 55,212‐2‐induced alterations in functional connectivity on this basis were thus defined as being significant increases or reductions in functional connectivity (where the value of the VIP statistic is significantly altered between the groups but the 95% CI of the VIP statistic exceeds the 0.8 threshold in both experimental groups), or significantly lost or newly gained functional connectivity (WIN 55,212‐2‐treated animals significantly different from controls and the 95% CI of the VIP statistic exceeds the 0.8 threshold in only one of the experimental groups).

### Statistical analysis

Data are expressed as means ± SEM. All data sets were tested for normality and analysed in Graphpad Prism 6 software (RRID:SCR_002798) or R (version 3.4.4, http://www.r-project.org, RRID: SCR_001905). A test for outliers was performed using the Graphpad Outlier Calculator. NORT data were analysed using two‐tailed, paired or unpaired Student's *t*‐tests, as appropriate for each condition and as indicated in the figure legends. Overt alterations in LCGU were statistically analysed by Student's *t*‐test, with discrete RoI treated as independent variables, as previously justified (Kelly and McCulloch 1987). Significance was set at *p* < 0.05 throughout.

## Results

### Chronic, intermittent WIN‐55,212‐2 administration disrupts recognition memory

**Figure 2:**
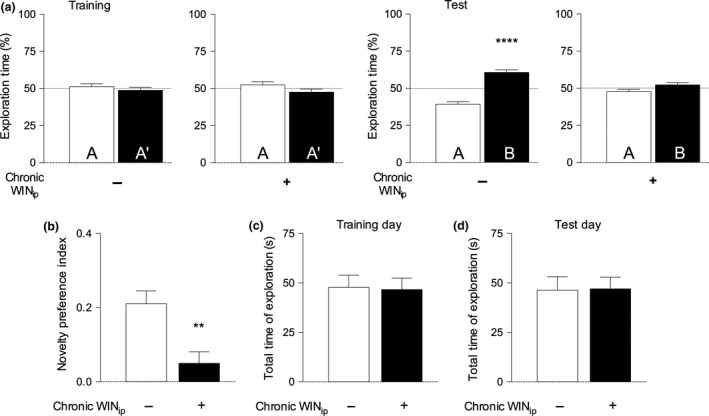
Chronic, intermittent WIN 55, 212‐2 treatment impairs recognition memory in the novel object recognition test (NORT). Panel a. Data are represented as % of exploration time. A denotes familiar object 1, A’ denotes familiar object 2, B denotes the novel object. – On the training day the % of time spent exploring each of the familiar objects did not differ between control and WIN 55,212‐2‐treated animals (*p* > 0.05, paired Student's *t*‐test). On the test day, control animals explored the novel object significantly more than the familiar object (****p < 0.0001, paired Student's *t*‐test). However, animals treated chronically with WIN 55,212‐2 spent a similar amount of time exploring both the familiar and novel object, (*p* > 0.05, paired Student's *t*‐test, comparing the % of time spent with a and b). Panel (b) – The Novelty Preference Index (NPI) is significantly decreased in WIN 55‐212‐2‐treated animals as compared with controls (**p < 0.01, unpaired Student's *t*‐test). A novelty index of zero represents absence of discrimination between objects (see Methods for details). The NPI of WIN55,212‐2‐treated mice was not significantly different from zero (*p* > 0.05, unpaired Student's *t*‐test), whereas in control mice the NPI was significantly different (*p* < 0.0001) from zero. Panels (c) and (d) – Total time of object exploration (TTE) during the training and test phase of novel object recognition test (NORT) was not significantly different between VEH and WIN‐treated animals (*p* > 0.05, unpaired Student's *t*‐test). An *a posteriori* Power Analysis for novel object recognition test (NORT) data was performed using G*power 3.1 software; considering a mean of 0.201 of preference for the novelty in control and 0.050 for drug‐treated animals (pooled SD (0.15), there is a 87.5% correct chance of correctly rejecting the null hypothesis (no differences on the *t*‐test), using 19 animals in the control group and 20 animals in the experimental group from a total of 39 animals.

Chronic, intermittent exposure to the cannabinoid agonist WIN 55,212‐2 disrupted recognition memory evaluated in the NORT. In the training phase, control animals (vehicle treated) and animals treated chronically with WIN 55,212‐2 explored the two identical objects for a similar amount of time (Fig. 2, panel a). However, on the test day, while control animals showed a significant preference for the novel as compared with the familiar object (*p* < 0.001, *n* = 19), WIN 22,515‐2‐treated animals did not (*p* > 0.05, *n* = 20). A similar effect was seen when analysing the novelty preference index (NPI) with control animals showing a significant preference for the novel object during the test phase (*p* < 0.05, *n* = 19), while WIN 55,212‐2‐treated animals did not (Fig. 2, panel b). Importantly, we found that there was no significant difference in the total time of object exploration between control and WIN 55,212‐2‐treated mice, during either the training or test phase (Fig. 2, panels c and d).

The NPI scores obtained in this work were not significantly different from zero, being similar to those previously reported by us using the same NORT paradigm in mice of the same age during acute WIN 55,212‐2 administration (Mouro *et al*. 2017). This indicates that the treatment schedule used in this work does not result in tolerance with regards to recognition memory. Once confirmed this was then taken as a positive control to proceed for the assessment of the influence of WIN 55,212‐2 upon brain metabolism and functional brain connectivity, to elucidated the functional changes underlying this deficit.

### Chronic, intermittent WIN‐55,212‐2 administration does not alter locomotor behaviour or anxiety‐like behaviour

Anxiety‐like behaviour and locomotor abilities were measured before, after and during the memory test, using, respectively, the OF test, the EPM and by measuring the total time of object exploration during the test phase of the NORT. We found no evidence to suggest that chronic WIN 55,212‐2 administration significantly impacted on locomotor activity or anxiety‐like behaviour (Figs 2 panel c and d and 3).

**Figure 3:**
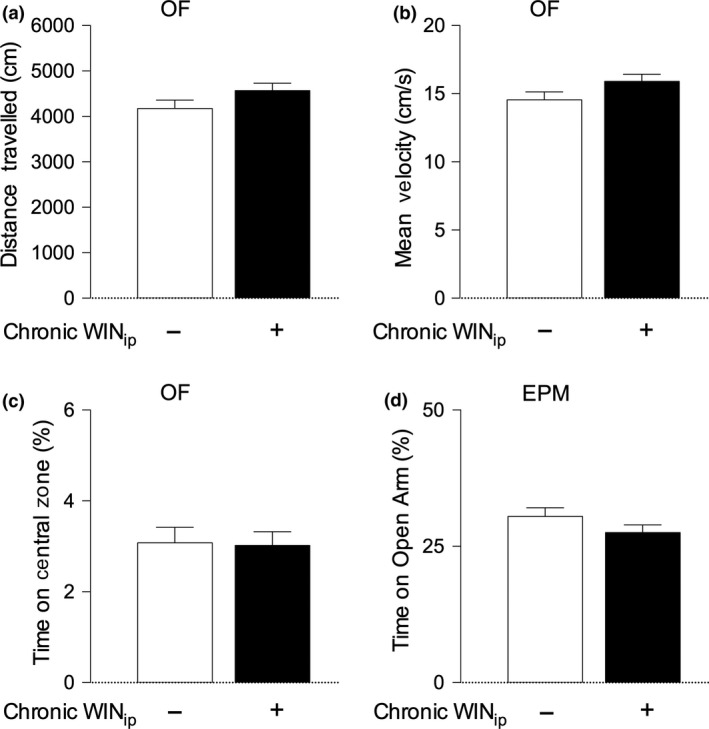
Chronic, intermittent WIN55, 212‐2 treatment does not impact on locomotor activity or anxiety‐like behaviour. Panel (a) and (b) – Locomotor abilities were assessed in the open field test (OFT) (panels (a) and (b)), performed during the first habituation day of the novel object recognition test (NORT). Chronic WIN, 55,212‐2 treatment did not significantly alter the total distance travelled (panel a) or velocity (panel b) in comparison with vehicle‐treated controls (*p* > 0.05, unpaired Student's *t*‐test). Panels (c) and (d) –Anxiety‐like behaviour was assessed at two time points: before the novel object recognition test (NORT) in the OFT, by evaluating the percentage of time spent in the central zone (panel c), and after novel object recognition test (NORT), in the EPM test (panel d) by evaluating the percentage of time spent in the open arms of the maze. In both tests, there were no significant differences between WIN 55,212‐2‐treated animals and saline‐treated controls (*p* > 0.05, unpaired Student's *t*‐test).

### Chronic, intermittent WIN 55,212‐2 administration alters LCGU

**Figure 4:**
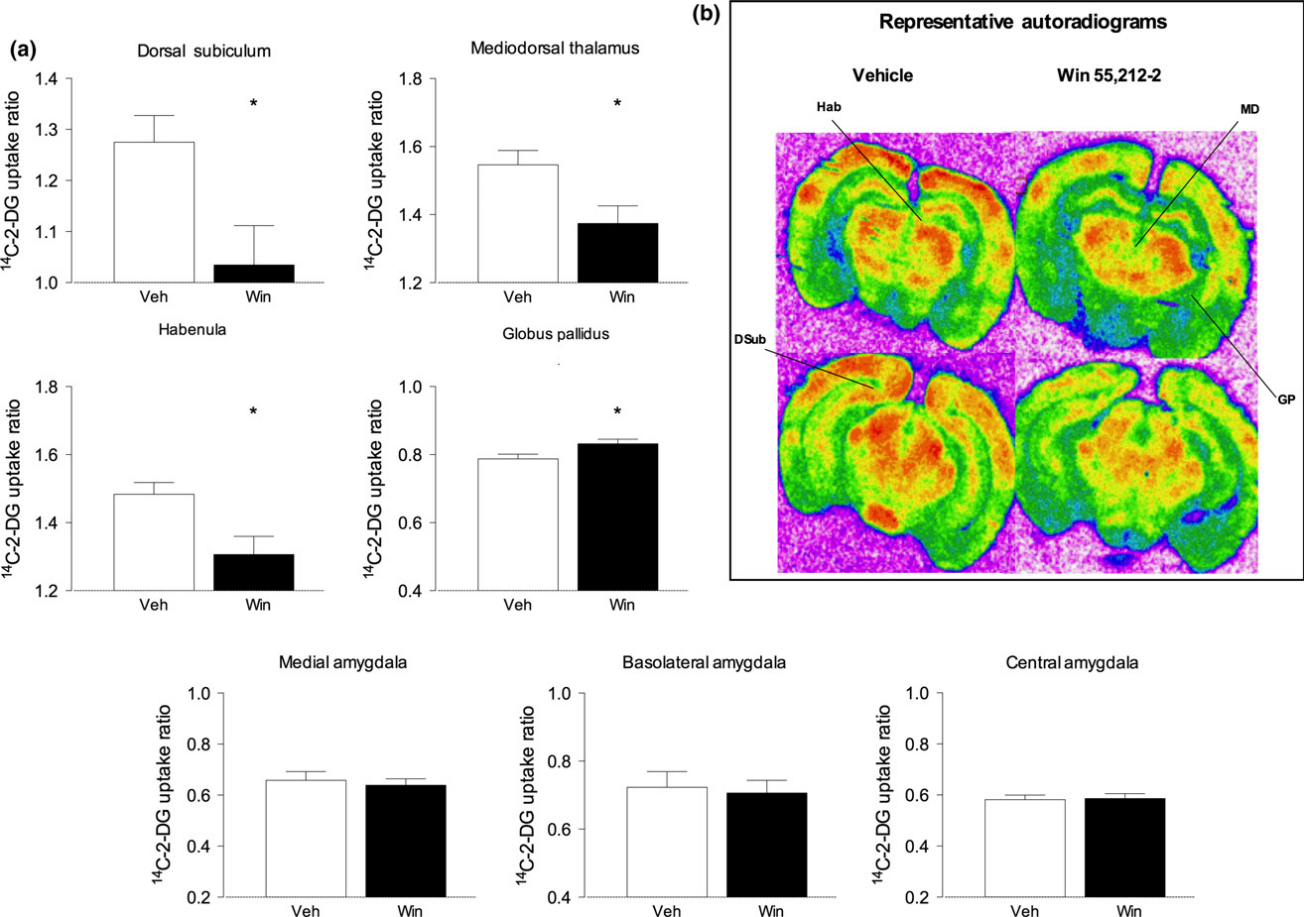
Chronic, intermittent WIN 55,212‐2 administration induces localized increases and decreases in local cerebral glucose utilization. Panel (a) – Chronic, intermittent WIN 55,212‐2 administration induced significantly decreased cerebral metabolism in the dorsal subiculum (DS), Mediodorsal thalamus (MD) and Habenula (Hab) and increased metabolism in the Globus Pallidus (GP). By contrast, cerebral metabolism in the amygdala nuclei (Basolateral, BLA; Central, CeA; Medial, MeA) was not significantly altered in WIN 55,212‐treated animals. | Data shown as the ^14^C‐2‐DG uptake ratio obtained for each RoI. *denotes *p* < 0.05 significant difference from control (unpaired Student's *t*‐test). Panel (b) – representative false colour autoradiograms at the level of the dorsal and ventral hippocampus showing altered cerebral metabolism in WIN 55,212‐2‐treated animals. Higher rates of metabolism are indicated by warmer colours (red/orange) and lower rates of metabolism by cooler colours (blue/green). Full data are shown in the Table S2.

Chronic, intermittent WIN 55,212‐2 administration induced both increases and decreases in LCGU on a brain region‐dependent basis. WIN 55,212‐2‐treated mice showed marked hypometabolism in the dorsal subiculum of the ventral hippocampus (DSub), the thalamic mediodorsal nucleus (MD) and in the habenula (Hab) (*p* < 0.05, *n* = 10). By contrast, WIN 55,212‐2‐treated animals showed significant hypermetabolism in only one of the brain regions analysed, the Globus Pallidus (GP) (*p* < 0.05, *n* = 10). These results support altered function in the basal ganglia–thalamic–hippocampal circuits (Fig. 4). Interestingly, when measuring LCGU on three different amygdala regions, there were no statistically significant differences between control and experimental groups. Thus, these data support the results obtained in the behaviour tests which showed no significant differences in anxiety‐like behaviour (Fig. 3) following chronic WIN 55,212‐2 administration. Full ^14^C‐2‐deoxyglucose brain imaging data are shown in the supplemental material (Table S2). Blood glucose and level of ^14^C‐2‐DG in the plasma were not significantly altered in control and WIN 55,212‐2‐treated animals (Table S1).

### Regional functional connectivity

**Figure 5:**
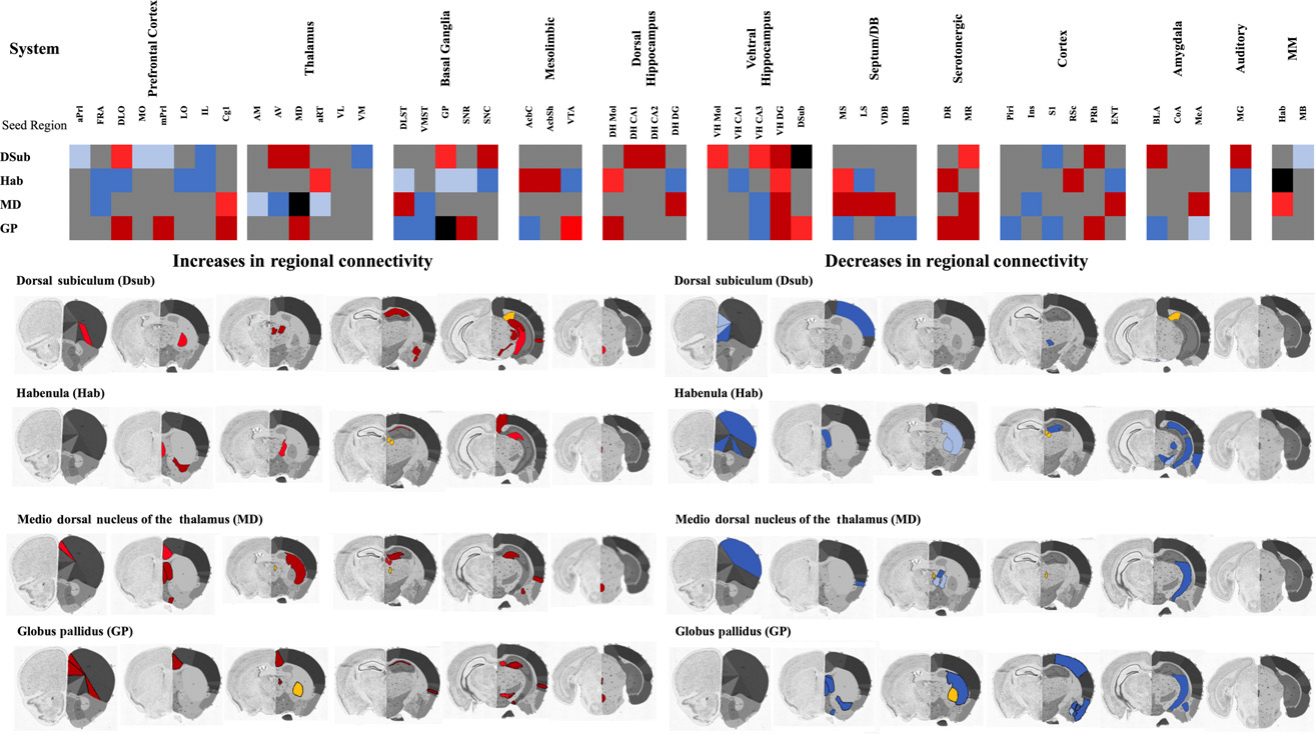
Chronic, intermittent WIN 55,212 treatment alters functional connectivity in neural circuits underpinning recognition memory. Panel (a) | Heatmap showing how chronic, intermittent treatment with WIN 55,212‐2‐induced modifications in the functional connectivity of ‘seed’ brain regions (DSub, MD, Hab and GP). Dark red denotes significantly gained connectivity, whereas light red represents significantly increased connectivity. Dark blue represents significantly lost connectivity, whereas light blue denotes significantly decreased connectivity. Significant gains, losses, increases and decreases in connectivity were determined by statistical comparison of the VIP statistic (unpaired Student's *t*‐test with Bonferroni *post hoc* correction) determined PSLR, with significance level set at *p* < 0.05. Full data for each ‘seed’ region are shown in the Tables S3–S6. Panel (b) | Brain images showing the anatomical localization of brain regions with significant increased/gained connectivity to the ‘seed’ regions (DSub, Hab, MD and GP). Yellow represents the anatomical localization of the ‘seed’ brain region. Brain section figures are modified from the Allen mouse brain atlas (mouse.brain‐map.org/static/atlas).

We analysed the functional connectivity of four ‘seed’ regions, defined as those where we had identified a significant impact of WIN 55,212‐2 treatment on LCGU (DSub, MD, Hab, GP). Analysis by means of PLSR algorithm revealed significant modifications in functional connectivity in the mice that had received WIN 55,212‐2 (Fig. 5).

For the DSub of the ventral hippocampus, in control animals this region was significantly connected to multiple subfields of the PFC, selected other ventral hippocampal subfields and to the serotonergic raphé (dorsal raphé, DR; median raphé, MR; Table S3). In animals treated with WIN 55,212‐2, functional connectivity of the DSub was significantly altered. Specifically, significant decreases in (anterior prelimbic, aPrL; medial orbital MO and medial prelimbic, mPrL) and lost (infralimbic, IL) connectivity with multiple PFC regions were seen in animals treated chronically with WIN 55,212‐2. By contrast, chronic WIN 55,212‐2 administration resulted in new abnormal functional connectivity between the DSub of the ventral hippocampus and subfields of the dorsal hippocampus (CA1, CA2). In addition, functional connectivity of the DSub to other subfields in the ventral hippocampus was significantly enhanced (Mol, CA3) or gained (DG) in WIN 55,212‐2‐treated animals. This suggests that the local connectivity of the ventral hippocampus DSub to other hippocampal subfields was significantly increased as a result of chronic WIN 55,212‐2 administration. In addition, functional connectivity of the DSub to the perirhinal cortex was abnormally gained in response to chronic WIN 55,212‐2 treatment. Furthermore, the DSub gained new functional connectivity to discrete thalamic nuclei (anteroventral, AV; mediodorsal, MD) and to the serotonergic MR following chronic WIN‐55,212‐2 administration.

In control animals the Hab was functionally connected with several PFC subfields and several striatal, hippocampal and amygdala regions (Table S4). Following chronic WIN 55,212‐2 treatment the functional connectivity of the Hab was significantly altered. On one hand, the Hab lost functional connectivity with multiple PFC subfields (frontal association area; dorsolateral orbital cortex; lateral orbital cortex, LO and IL) and showed significantly lost (substantia nigra pars compacta, SNC) and decreased connectivity (dorsolateral striatum; GP and substantia nigra pars reticulata, SNR) to several regions of the basal ganglia. By contrast, WIN 55,212‐2 treatment provoked abnormal gains in connectivity between the Hab and subfields of the nucleus accumbens (core, AcbC; shell, AcbSh), with the serotonergic system (dorsal raphé, DR).

In control animals the MD thalamic nucleus was significantly functionally connected to multiple subfields of the prefrontal cortex (PFC), other cortical regions and other thalamic nuclei (Table S5). The functional connectivity of the MD was significantly altered in mice treated chronically with WIN 55,212‐2. This included significantly lost (anteroventral nucleus, AV) or decreased (anteromedial nucleus, AM; anterior reticular thalamus) connectivity to other thalamic nuclei, and cortical regions (frontal association cortex, frontal association area; insular cortex, Ins). By contrast, chronic WIN 55,212‐2 treatment resulted in new, abnormal functional connectivity between the MD and multiple subfields of the septum/diagonal band of Broca (medial septum, MS; lateral septum, LS; ventral limb of the diagonal band of broca, VDB).

Finally, in control animals the GP was functionally connected to numerous regions of the septum/diagonal band of Broca, and to striatal and cortical regions (Table S6). As a consequence of chronic WIN 55,212‐2 treatment the functional connectivity of the GP was significantly modified. The GP significantly lost functional connectivity with several regions of the septum/diagonal band of Broca (medial septum, MS; ventral limb diagonal band of Broca, VDB; horizontal limb diagonal band of Broca) and to the striatum (dorsolateral striatum and ventrolateral striatum). By contrast, the GP gained abnormal new connectivity with PFC (dorsolateral orbital cortex, mPrl and cingulate cortex, Cg1) and to the serotonergic raphe (MR and DR) following chronic, intermittent WIN 55,212‐2 treatment.

## Discussion

In this work we demonstrated that mice chronically exposed to the non‐selective cannabinoid receptor agonist, WIN 55,212‐2, displayed disrupted cerebral metabolism and abnormal functional connectivity in the cortico–thalamic–hippocampal circuits that underlie recognition memory. This includes compromised perirhinal–hippocampus–prefrontal cortex and thalamo‐prefrontal functional connectivity. In parallel, we observed deficits in recognition memory as a consequence of chronic WIN 55,212‐2 administration without signs of altered motor abilities and anxiety‐like behaviour.

CB_1_Rs on synapses inhibit glutamatergic and GABAergic transmission, modulate different forms of synaptic plasticity and control neural oscillations that support behaviour and diverse cognitive functions, including learning and memory (Hajos *et al*. 2000; Piomelli 2003; Kano *et al*. 2009; Albayram *et al*. 2016; Araque *et al*. 2017; Lupica *et al*. 2017). Altogether, previous data also suggest that endo‐ and exo‐ cannabinoids may induce the functional reconfiguration of neuronal and brain networks to impact on memory processing, and we now specifically addressed this possibility.

Object recognition learning and memory is a process involving multiple items, the contextual clues surrounding them and the temporal order in each they are presented. Effective recognition memory depends on functional interactions within a circuit comprised by the perirhinal cortex (Bussey *et al*. 2000; Warburton and Brown 2010), the hippocampus (Brown and Aggleton 2001; Barker and Warburton 2011), the medial prefrontal cortex (O'Neil *et al*. 2012) and the mediodorsal nucleus of the thalamus (Mitchell *et al*. 2005; Warburton and Brown 2015). In this work we report deficits on recognition memory following chronic, intermittent WIN 55,212‐2 treatment as assessed by the NORT. Data from the behavioural test served as a positive confirmation for the results obtained from the brain imaging and functional connectivity experiments, discussed below. We found that chronic, intermittent WIN 55‐212‐2 administration significantly impacts on the function and connectivity of the hippocampal dorsal subiculum (DSub, Fig. 5), in line with previous suggestions that the subiculum is involved in recognition memory (Chang and Huerta 2012). The subiculum is a primary output structure of the hippocampus and receives direct projections from other brain regions critical for recognition memory, including the perirhinal cortex (Amaral *et al*. 2007). Monosynaptic and reciprocal connections between the subiculum and the perirhinal and the postrhinal cortices exist (Witter *et al*. 2000), implicating the subiculum in a short functional loop with cortical areas known to be crucial for recognition memory (Warburton and Brown 2010). Our study revealed that chronic, intermittent WIN 55,212‐2 treatment induced a pattern of irregular and dysfunctional connectivity between the dorsal subiculum and virtually every other subfield of the hippocampus (CA1, CA2, CA3, ML and DG; Fig. 5). Furthermore, chronic, intermittent WIN 55,212‐2 treatment impaired connectivity between the dorsal subiculum and several subfields of the prefrontal cortex, another structure directly implicated in recognition memory (Riba *et al*. 2015).

We also found widespread evidence for functional dysconnectivity of the mediodorsal (MD) thalamic nuclei as a consequence of WIN 55,212‐2 administration, which could also contribute to the deficits in recognition memory seen in these animals. Indeed, there is building evidence supporting a cortico–thalamic–hippocampal network, including the MD, that underlies recognition memory, in part as a result of the ability of the mediodorsal thalamic nuclei to act as a relay between the perirhinal and the medial prefrontal cortex (Parker and Gaffan 1998; Warburton and Brown 2015). The mediodorsal thalamic nucleus directly projects to the hippocampus and to the prefrontal cortex. Lesions in this thalamic nucleus result in recognition memory deficits (Parker *et al*. 1997), replicating deficits related with lesions of the medial temporal lobe (Warburton and Brown 2015). In addition, it has been previously shown that disconnection of the mediodorsal thalamic nucleus from the medial temporal cortex impairs object‐in‐place and temporal order performance (Cross *et al*. 2012).

The habenula is an important anatomical hub involved in a diverse range of behaviours including reward processing, reward prediction error, memory and the stress response (Naamboodiri *et al*. 2016). The habenula receives direct projections from the prefrontal cortex and the basal ganglia (globus pallidus, Hikosaka *et al*. 2008), regions that show reduced functional connectivity to the habenula after chronic, intermittent WIN 55,212‐2 administration (Fig. 4). The habenula also sends direct projections to dopamine‐rich brain regions including the ventral tegmental area and substantia nigra pars compacta (SNC, Hikosaka *et al*. 2008). Remarkably, the functional connectivity of the habenula to these regions is also lost in animals treated chronically with WIN 55,212‐2. Moreover, the functional connectivity of the habenula to other components of the mesolimbic system, including the nucleus accumbens, is also significantly altered by chronic WIN 55,212‐2‐treated animals. These effects may relate to the amotivation syndrome (Tunving 1987) and reward processing deficits (Fujiwara 2001; Friemel *et al*. 2014) seen as a result of cannabinoid exposure. By contrast, the functional connectivity of the habenula to the serotonergic raphe, to which the habenula directly projects, is abnormally enhanced by chronic WIN 55,212‐2 administration. This suggests that chronic cannabinoid exposure may impact both dopaminergic and serotonergic system function by impacting, in part, on the functional connectivity of the habenula.

Altered serotonin system function as a result of chronic, intermittent WIN 55,212‐2 administration, is also supported by broader evidence of altered raphé nuclei functional connectivity, being evident to each of the seed brain regions analysed. A number of previous studies have found that chronic cannabinoid exposure alters the functioning of the serotonin system, including the induction of altered serotonin levels (Sagredo *et al*. 2006) and serotonin receptor activity (Darmani 2001; Hill *et al*. 2006; Moranta *et al*. 2009; Franklin *et al*., 2013; Esteban and García‐Sevilla 2012). Moreover, the role of the serotonin system in recognition memory is firmly established (Zhang *et al*. 2013, 2015) suggesting that the disruption of serotonin system connectivity may be one of the key mechanisms by which chronic, intermittent WIN 55,212‐2 exposure impairs recognition memory. This possibility warrants, however, further systematic investigation. If found to be correct, targeting the serotonin system may represent a therapeutic strategy to restore memory deficits as a consequence of chronic cannabinoid exposure.

Cannabinoid intake induces more severe behavioural deficits in pubertal rats than in mature animals (Schneider and Koch 2002; Schneider *et al*., 2008). Indeed, short‐term recognition memory impairments (30 min retention time in NORT) can persist beyond cannabinoid exposure if the exposure occurs during the pubertal period (Schneider and Koch 2002; Schneider *et al*., 2008), but not if the exposure occurs only during adulthood (Schneider and Koch 2003) or even if it occurs only during the pre‐pubertal period (Schneider *et al*. 2005). However, as we herein show, cannabinoid exposure in adulthood does induce alterations in brain metabolism and connectivity, and these alterations are accompanied by significant recognition memory impairments over longer retention intervals (24 h). In addition, the present evidence showing that functional connectivity between the thalamus and prefrontal cortex is affected by cannabinoid exposure is in line with previous findings that cannabinoids can exacerbate deficits induced by prefrontal cortex lesions (Schneider and Koch 2005; Schneider and Koch 2007).

The exact pharmacological identification of the receptor influenced by the cannabinoid compound used in this work is outside its objective. However, there is evidence that supports the possibility of the presently described actions of WIN 55,212‐2 being mediated through CB_1_R. WIN 55‐212,2 is one of the most commonly used cannabinomimetics to study the role of the CB_1_R and the CB_2_R (Solymosi and Köfalvi 2017), showing similar preference for activating both receptors (Pertwee *et al*. 2010). On the other hand, evidence has consistently shown that WIN 55,212‐2 has no effect upon G protein‐coupled receptor 55 (GPR55) (Johns *et al*. 2007; Ryberg *et al*. 2007; Pertwee *et al*. 2010; Solymosi and Köfalvi 2017). We previously showed that the CB_1_R selective antagonist (AM 251) abolished the impact of acute WIN 55,212‐2 administration on recognition memory, which was evaluated with the same NORT paradigm and using mice of the same age as used in this study (Mouro *et al*. 2017), supporting involvement of CB_1_R. There is, in fact, a broad range of evidence supporting the involvement of CB_1_R in memory deficits (Clarke *et al*. 2008; Suenaga and Ichitani 2008; Wise *et al*. 2009), while CB_2_R agonists do not seem to affect recognition memory (Clarke *et al*. 2008). Regarding modifications of brain metabolism following cannabinoid administration, evidence shows that CB_1_R agonists lead to decreases in glucose uptake (Duarte *et al*. 2012; Miederer *et al*. 2017) and mitochondrial respiration (Bénard *et al*. 2012).

In conclusion, we herein demonstrated that prolonged, intermittent exposure of adult mice to the non‐selective cannabinoid receptor agonist WIN 55,212‐2 induces alterations in metabolic brain activity in selected brain regions, alters their functional connectivity and impairs recognition memory. Connectivity modifications were seen in circuits known to be directly involved in recognition memory, and for the habenula and raphé nuclei. These data give new insight into the mechanisms by which chronic cannabinoid exposure impacts on behaviour and cognition, and highlight the value of considering cannabinoid actions at the systems‐level perspective.

## Author contributions

FMM conducted the experiments and data acquisition, as well as performed all habituation procedures and data analysis. ND contributed to data acquisition, to the design of the brain connectivity experiments and introduced FMM to the corresponding technical procedures and data analysis. AMS and JAR contributed to data discussion. AMS designed the project. FMM, AMS and ND wrote the manuscript. All authors contributed to the final version of the manuscript.

## Acknowledgments and conflict of interest disclosure

This work was supported by LISBOA‐01‐0145‐FEDER‐007391, project co‐funded by FEDER through POR Lisboa 2020 (Programa Operacional Regional de Lisboa) from PORTUGAL 2020 and Fundação para a Ciência e Tecnologia (FCT), by an FCT project (Fundação para a Ciência e a Tecnologia, PTDC/DTP‐FTO/3346/2014) and by a Twinning action (SynaNet) from the EU H2020 Programme (project number: 692340), which covered short‐term scientific missions of FMM at ND laboratory and of ND at AMS laboratory. FMM was in receipt of SFRH/BD/89582/2012 FTC fellowship. AMS is a handling editor for the Journal of Neurochemistry.

All experiments were conducted in compliance with the ARRIVE guidelines.

## Supporting information


**Table S1.** Body weight and blood parameters for animals undergoing the 14C‐2‐DG experiment.
**Table S2.** Local cerebral glucose utilization (LCGU) in WIN 55,212‐2 and control animals.
**Table S3.** Functional Connectivity of the Ventral Hippocampal Dorsal Subiculum.
**Table S4**. Functional Connectivity of the Habenula.
**Table S5.** Functional Connectivity of the Mediodorsal Thalamic Nucleus.
**Table S6.** Functional Connectivity of the Globus Pallidus.
