# Supplementary material for: Chronic, intermittent treatment with a cannabinoid receptor agonist impairs recognition memory and brain network functional connectivity
Source: J Neurochem. 2018 Sep 27;147(1):71–83. doi: 10.1111/jnc.14549 (PMC6220860; doi:10.1111/jnc.14549)
Supplement: Supplementary file 1 — Table S1. Body weight and blood parameters for animals undergoing the 14C‐2‐DG experiment. Table S2. Local cerebral glucose utilization (LCGU) in WIN 55,212‐2 and control animals. Table S3. Functional Connectivity of the Ventral Hippocampal Dorsal Subiculum. Table S4. Functional Connectivity of the Habenula. Table S5. Functional Connectivity of the Mediodorsal Thalamic Nucleus. Table S6. Functional Connectivity of the Globus Pallidus. [file JNC-147-71-s001.pdf]

Supplementary Material

Chronic, intermittent treatment with a cannabinoid receptor agonist impairs recognition memory and brain network functional connectivity

Francisco M Mouro[1,2], Joaquim A Ribeiro[1,2], Ana M Sebastião[1,2*], Neil Dawson[3*]

[1] Instituto de Farmacologia e Neurociências, Faculdade de Medicina, Universidade de Lisboa, Portugal

[2] Instituto de Medicina Molecular, Faculdade de Medicina, Universidade de Lisboa, Portugal

[3] Division of Biomedical and Life Sciences, University of Lancaster, United Kingdom

*co-senior authors

Corresponding author: AM Sebastião, Instituto de Farmacologia e Neurociências, Faculdade de Medicina, Av. Prof Egas Moniz, 1649-028 Lisboa, Portugal. Email: anaseb@medicina.ulisboa.pt; tel: +351-21-7985183

Table S1. Body weight and blood parameters for animals undergoing the 14C-2-DG experiment

Treatment group	Weight (g)	^{14}C-2-DG in plasma (nCi/ml)	T-Test (diff)	Blood Glucose (nmol/L)	T-Test (diff)
**Vehicle**	25.87 ± 0.53	0.95 ± 0.16	0.55, n.s	11.12 ± 0.36	0.74, n.s
**WIN 55,212-2**	26.89 ± 0.35	1.36 ± 0.65		10.96 ± 0.33	0.16 ± 0.50

Chronic, intermittent WIN 55-212-2 administration did not significantly alter the weight of the animals. Blood glucose levels and 14C-2-DG levels at the moment of animal sacrifice show no significant statistical differences between WIN 55-212-2 treated animals and controls (n = 10, under unpaired Student's T Test).

Table S2. Local cerebral glucose utilisation (LCGU) in WIN 55,212-2 and control animals

RoI	Full name	Mean Veh ^{14}C-2-DG UR	SE	Mean Win ^{14}C-2-DG UR	SE	T-test P value
aPrL	Anterior Prelimbic Cortex	1.175	3.27	1.196	2.70	0.682
FRA	Frontal Assotiation Area	1.086	2.33	1.110	1.51	0.433
DLO	Dorsolateral Orbital Cortex	0.852	2.20	0.905	3.56	0.157
LO	Lateral Orbital Cortex	1.390	3.71	1.441	4.72	0.540
MO	Medial Orbital Cortex	1.119	3.54	1.145	5.24	0.715
mPrL	Medial Prelimbic Cortex	1.097	5.03	1.117	3.07	0.758
IL	Infralimbic Cortex	0.946	6.25	0.976	3.72	0.674
AcbC	Nucleus Accumbens Core	0.880	2.40	0.900	1.86	0.442
AcbSh	Nucleus Accumbens Shell	0.903	1.99	0.910	1.75	0.772
DLST	Dorsolateral Striatum	1.249	2.06	1.249	1.64	0.994
VMST	Ventrolateral Striatum	1.193	2.14	1.178	1.72	0.646
MS	Medial Septum	1.010	3.28	0.958	3.12	0.245
LS	Lateral Septum	0.830	1.71	0.789	4.21	0.249
VDB	Ventral Limb of Diagonal Band of Broca	1.056	4.27	0.968	3.78	*0.139*
HDB	Horizontal Limb of Diagonal Band of Broca	0.854	3.76	0.908	3.65	0.238
Piri	Piriform Cortex	1.299	2.14	1.238	2.65	0.157
Ins	Insular Cortex	0.769	2.23	0.774	2.29	0.844
Cg1	Cingulate Cortex	1.069	2.97	1.105	2.57	0.399
S1	Somatosensory Cortex	1.196	2.89	1.256	1.95	0.164
AV	Anteroventral Thalamus	1.479	6.12	1.491	5.90	0.927
AM	Anteromedial Thalamus	1.242	5.91	1.166	7.05	0.508
aRT	Reticular Thalamus	1.504	6.86	1.359	6.86	0.328
**GP**	Globus Pallidus	**0.787**	**1.68**	**0.832**	**1.75**	**0.038***
**MD**	Mediodorsal Thalamus	**1.546**	**2.72**	**1.374**	**3.96**	**0.019***
VL	Ventrolateral Thalamus	1.689	3.78	1.580	2.00	0.139
**Hab**	Habenula	**1.484**	**2.32**	**1.307**	**4.30**	**0.012***
VM	Ventromedial Thalamus	1.312	4.15	1.315	2.74	0.958
BLA	Basolateral Amygdala	0.723	6.43	0.706	5.60	0.786
CoA	Central Amygdala	0.582	3.04	0.586	3.49	0.878
MeA	Medial Amygdala	0.658	5.18	0.639	3.89	0.658
RSc	Retrosplenial Cortex	1.473	3.01	1.375	3.17	0.133
DH Mol	Dorsal Hippocampus, Molecular Layer	1.427	2.03	1.410	2.91	0.729
DH CA1	Dorsal Hippocampus, CA1	0.802	2.35	0.770	2.88	0.268
DH CA2	Dorsal Hippocampus, CA2	0.716	1.54	0.731	2.22	0.442
DH DG	Dorsal Hippocampus, Dentate Gyrus	0.764	3.17	0.845	5.01	0.098
MG	Medial Geniculate	1.584	3.44	1.582	2.46	0.972
VH LMol	Ventral Hippocampus, Molecular Layer	1.255	2.33	1.191	3.65	0.222
**DSub**	Dorsal Subiculum	**1.275**	**3.89**	**1.035**	**7.85**	**0.022***
VH CA1	Ventral Hippocampus, CA1	0.982	6.30	0.905	3.01	0.264
VH CA3	Ventral Hippocampus, CA3	0.712	4.13	0.711	1.93	0.968
VH DG	Ventral Hippocampus, Dentate Gyrus	0.700	5.33	0.753	2.74	0.225
SNR	Substancia Nigra pars Reticulata	0.757	5.75	0.745	5.77	0.850
PRh	Perirhinal Cortex	0.880	2.68	0.911	3.09	0.404
ENT	Entorhinal Cortex	0.729	2.09	0.757	1.99	0.207
SNC	Substancia Nigra pars Compacta	0.889	3.62	0.885	5.99	0.950
MB	Mammillary Body	1.745	3.49	1.616	7.96	0.405
VTA	Ventral Tegmental Area	1.254	5.19	1.291	6.63	0.732
DR	Dorsal Raphe	0.892	4.19	0.821	4.62	0.203
MR	Medial Raphe	0.988	4.95	0.949	6.40	0.620

Impact of chronic, intermittent WIN 55,212-2 administration on local cerebral glucose utilisation (LCGU). Data is shown as Mean ± SEM ^{14}C-2-DG uptake ratio. In bold, statistically significant differences found between WIN 55,212-2 and vehicle treated animals are shown. *denotes $p<0.05$, Student's t-test.

Table S3: Functional Connectivity of the Ventral Hippocampal Dorsal Subiculum

RoI	Control			WIN55,212-2 Treated		
anterior Prelimbic Cortex (aPrL)	**1.57**	±	**0.03**	**1.13****	±	**0.06**
Frontal Association Cortex (FRA)	**1.30**	±	**0.06**	**1.38**	±	**0.02**
Dorsolateral Orbital Cortex (DLO)	**0.99**	±	**0.04**	**1.41****	±	**0.06**
Lateral Orbital Cortex (LO)	**0.92**	±	**0.07**	0.65	±	0.04
Medial Orbital Cortex (MO)	**1.99**	±	**0.04**	**0.94*****	±	**0.05**
medial Prelimbic Cortex (mPrL)	**1.63**	±	**0.04**	**0.97*****	±	**0.07**
Infralimbic Cortex (IL)	**1.49**	±	**0.03**	0.47***	±	0.05
Nucleus Accumbens Core (AcbC)	0.64	±	0.07	0.64	±	0.05
Nucleus Accumbens Shell (AcbSh)	0.41	±	0.05	0.47	±	0.04
Dorsolateral Striatum (DLST)	0.48	±	0.06	0.70	±	0.05
Ventromedial Striatum (VMST)	**0.99**	±	**0.06**	**1.16**	±	**0.04**
Medial Septum (MS)	0.46	±	0.04	0.77**	±	0.04
Lateral Septum (LS)	0.45	±	0.05	0.36	±	0.03
Ventral Limb Diagonal Band Broca (VDB)	**0.86**	±	**0.05**	**0.82**	±	**0.06**
Horizontal Limb Diagonal Band of Broca (HDB)	0.44	±	0.05	0.29	±	0.06
Piriform Cortex (Piri)	**1.02**	±	**0.10**	**0.92**	±	**0.07**
Insular Cortex (Ins)	0.49	±	0.05	0.34	±	0.09
Cingulate Cortex (Cg1)	**1.63**	±	**0.03**	**1.42**	±	**0.05**
Somatosensory Cortex (S1)	**1.46**	±	**0.10**	0.76***	±	0.03
Anteroventral Nucleus (AV)	0.39	±	0.03	**1.01*****	±	**0.08**
Anteromedial Nucleus (AM)	0.61	±	0.06	0.57	±	0.07
anterior Reticular Thalamus (aRT)	0.81	±	0.06	0.49	±	0.07
Globus Pallidus (GP)	**1.19**	±	**0.07**	**1.82*****	±	**0.03**
Mediodorsal Nucleus (MD)	0.61	±	0.05	**1.07****	±	**0.07**
Ventrolateral Nucleus (VL)	0.67	±	0.07	0.69	±	0.06
Habenula (Hab)	**0.87**	±	**0.10**	0.72	±	0.07
Ventromedial Nucleus (VM)	**1.28**	±	**0.03**	0.49***	±	0.06
Basolateral (BLA)	0.37	±	0.05	**1.06*****	±	**0.06**
Central (CeA)	0.60	±	0.07	**0.93**	±	**0.04**
Medial (MeA)	0.65	±	0.09	0.53	±	0.02
Retrosplenial Cortex (RSC)	0.54	±	0.13	0.49	±	0.06
Molecular Layer (DH.Mol)	0.63	±	0.07	0.57	±	0.07
Cornu Ammonis 1 (DH.CA1)	0.47	±	0.13	**1.56*****	±	**0.02**
Cornu Ammonis 2 (DH.CA2)	0.79	±	0.05	**1.21***	±	**0.07**
Dentate Gyrus (DH.DG)	0.76	±	0.03	**0.86**	±	**0.06**
Medial Geniculate (MG)	0.58	±	0.03	**1.18*****	±	**0.04**
Molecular Layer (VH.Mol)	**1.24**	±	**0.05**	**0.83***	±	**0.07**
Cornu Ammonis 1 (VH.CA1)	**1.48**	±	**0.05**	**1.53**	±	**0.02**
Cornu Ammonis 3 (VH.CA3)	**1.55**	±	**0.11**	**0.99***	±	**0.06**
Dentate Gyrus (VH.DG)	0.59	±	0.02	**0.86***	±	**0.05**
Substantia Nigra pars Reticulata (SNR)	0.66	±	0.06	**0.84**	±	**0.06**
Perirhinal Cortex (PRh)	0.53	±	0.08	**1.58*****	±	**0.03**
Entorhinal Cortex (EntC)	0.68	±	0.03	0.56	±	0.05
Substantia Nigra pars Compacta (SNC)	0.68	±	0.06	**1.06***	±	**0.05**
Mamillary Body (MB)	**1.34**	±	**0.05**	**0.85****	±	**0.07**
Ventral Tegmental Area (VTA)	**1.15**	±	**0.07**	**1.43**	±	**0.04**
Dorsal Raphé (DR)	**0.90**	±	**0.09**	**0.91**	±	**0.06**
Median Raphé (MR)	**0.89**	±	**0.05**	**1.48****	±	**0.08**

Functional connectivity of the dorsal subiculum (VH.DS) and its modification by chronic, intermittent WIN55,212-2 administration. Data shown represent the variable importance to the projection (VIP) as derived from partial least squares regression (PLSR) analysis with

the DSub as the “seed” brain region (dependent variable) and all other brain regions of interest (RoI) as the explanatory variables. Data were statistically analysed using Student’s t-test with Bonferroni post-hoc correction for multiple comparisons. Bold denote a significant functional connection to the seed region (95% confidence interval of the VIP statistic exceeds 0.8 threshold). *denotes $p<0.05$, **denotes $p<0.01$ and ***denotes $p<0.001$ significant difference from control animals. DH denotes dorsal hippocampus, VH denotes ventral hippocampus.

Table S4. Functional Connectivity of the Habenula

RoI	Control			WIN55,212-2 Treated		
anterior Prelimbic Cortex (aPrL)	1.11	±	0.07	0.74	±	0.08
Frontal Association Cortex (FRA)	1.26	±	0.11	0.74*	±	0.04
Dorsolateral Orbital Cortex (DLO)	1.02	±	0.06	0.47***	±	0.05
Lateral Orbital Cortex (LO)	1.05	±	0.05	0.64***	±	0.06
Medial Orbital Cortex (MO)	0.78	±	0.07	0.53	±	0.06
medial Prelimbic Cortex (mPrL)	1.01	±	0.03	0.89	±	0.07
Infralimbic Cortex (IL)	1.24	±	0.04	0.80***	±	0.05
Nucleus Accumbens Core (AcbC)	0.75	±	0.06	1.51***	±	0.05
Nucleus Accumbens Shell (AcbSh)	0.69	±	0.05	1.30***	±	0.06
Dorsolateral Striatum (DLST)	1.57	±	0.04	1.15	±	0.09
Ventromedial Striatum (VMST)	0.83	±	0.03	0.94	±	0.09
Medial Septum (MS)	1.03	±	0.03	1.670***	±	0.05
Lateral Septum (LS)	1.24	±	0.08	0.53***+	±	0.07
Ventral Limb Diagonal Band Broca (VDB)	1.49	±	0.06	1.50	±	0.07
Horizontal Limb Diagonal Band of Broca (HDB)	0.44	±	0.04	0.38	±	0.05
Piriform Cortex (Piri)	0.64	±	0.17	0.89	±	0.05
Insular Cortex (Ins)	0.97	±	0.08	1.09	±	0.06
Cingulate Cortex (Cg1)	1.07	±	0.05	0.92	±	0.06
Somatosensory Cortex (S1)	0.75	±	0.08	0.72	±	0.02
Anteroventral Nucleus (AV)	0.52	±	0.09	0.58	±	0.06
Anteromedial Nucleus (AM)	0.79	±	0.04	0.75	±	0.06
anterior Reticular Thalamus (aRT)	0.92	±	0.08	1.36*	±	0.05
Globus Pallidus (GP)	1.13	±	0.02	0.83*	±	0.06
Mediodorsal Nucleus (MD)	0.89	±	0.06	1.31	±	0.08
Ventrolateral Nucleus (VL)	0.78	±	0.05	0.80	±	0.05
Ventromedial Nucleus (VM)	0.83	±	0.07	0.88	±	0.02
Basolateral (BLA)	0.58	±	0.06	0.64	±	0.06
Central (CeA)	1.05	±	0.06	0.97	±	0.07
Medial (MeA)	0.93	±	0.06	0.91	±	0.06
Retrosplenial Cortex (RSC)	0.45	±	0.05	1.57***	±	0.03
Molecular Layer (DH.Mol)	0.94	±	0.04	2.07***	±	0.03
Cornu Ammonis 1 (DH.CA1)	0.53	±	0.05	0.58	±	0.06
Cornu Ammonis 2 (DH.CA2)	0.78	±	0.09	0.66	±	0.06
Dentate Gyrus (DH.DG)	1.20	±	0.08	0.68*	±	0.07
Medial Geniculate (MG)	1.22	±	0.04	0.51***	±	0.08
Molecular Layer (VH.Mol)	0.60	±	0.05	0.97	±	0.11
Dorsal Subiculum (VH.DS)	0.93	±	0.09	0.68	±	0.06
Cornu Ammonis 1 (VH.CA1)	1.25	±	0.03	0.69**	±	0.09
Cornu Ammonis 3 (VH.CA3)	1.00	±	0.08	1.07	±	0.08
Dentate Gyrus (VH.DG)	1.03	±	0.04	1.47**	±	0.06
Substantia Nigra pars Reticulata (SNR)	1.30	±	0.04	0.91***	±	0.04
Perirhinal Cortex (PRh)	0.68	±	0.03	0.50	±	0.09
Entorhinal Cortex (EntC)	0.93	±	0.04	0.39***	±	0.03
Substantia Nigra pars Compacta (SNC)	1.07	±	0.05	0.56**	±	0.08
Mamillary Body (MB)	0.39	±	0.05	0.45	±	0.04
Ventral Tegmental Area (VTA)	1.59	±	0.04	0.63***	±	0.11
Dorsal Raphé (DR)	0.51	±	0.07	1.15***	±	0.06
Median Raphé (MR)	1.14	±	0.12	1.34	±	0.06

Functional connectivity of the habenula (Hab) and its modification by chronic, intermittent WIN55,212-2 administration. Data shown represent the variable importance to the

projection (VIP) as derived from partial least squares regression (PLSR) analysis with the Hab as the "seed" brain region (dependent variable) and all other brain regions of interest (RoI) as the explanatory variables. Data were statistically analysed using Student's t-test with Bonferroni post-hoc correction for multiple comparisons. Bold denote a significant functional connection to the seed region (95% confidence interval of the VIP statistic exceeds 0.8 threshold). *denotes $p<0.05$, **denotes $p<0.01$ and ***denotes $p<0.001$ significant difference from control animals. DH denotes dorsal hippocampus, VH denotes ventral hippocampus.

Table S5. Functional Connectivity of the Mediodorsal Thalamic Nucleus

RoI	Control			WIN55,212-2 Treated		
anterior Prelimbic Cortex (aPrL)	**0.95**	±	**0.06**	0.56	±	0.10
Frontal Association Cortex (FRA)	**0.84**	±	**0.05**	0.34***	±	0.03
Dorsolateral Orbital Cortex (DLO)	**1.01**	±	**0.07**	0.83	±	0.13
Lateral Orbital Cortex (LO)	**0.87**	±	**0.09**	**0.95**	±	**0.03**
Medial Orbital Cortex (MO)	**0.90**	±	**0.08**	0.62	±	0.04
medial Prelimbic Cortex (mPrL)	**1.19**	±	**0.03**	**1.06**	±	**0.04**
Infralimbic Cortex (IL)	**1.31**	±	**0.05**	**1.41**	±	**0.09**
Nucleus Accumbens Core (AcbC)	0.47	±	0.04	0.76	±	0.03
Nucleus Accumbens Shell (AcbSh)	**1.18**	±	**0.09**	**1.11**	±	**0.04**
Dorsolateral Striatum (DLST)	0.74	±	0.04	**1.13***	±	**0.08**
Ventromedial Striatum (VMST)	**1.54**	±	**0.08**	0.65***	±	0.09
Medial Septum (MS)	0.57	±	0.11	**1.58*****	±	**0.06**
Lateral Septum (LS)	0.54	±	0.07	**1.09*****	±	**0.04**
Ventral Limb Diagonal Band Broca (VDB)	0.71	±	0.07	**1.13***	±	**0.05**
Horizontal Limb Diagonal Band of Broca (HDB)	0.58	±	0.07	0.37	±	0.07
Piriform Cortex (Piri)	0.53	±	0.06	0.29	±	0.05
Insular Cortex (Ins)	**1.37**	±	**0.05**	0.39***	±	0.09
Cingulate Cortex (Cg1)	**1.03**	±	**0.10**	**1.55***	±	**0.06**
Somatosensory Cortex (S1)	0.68	±	0.06	0.66	±	0.06
Anteroventral Nucleus (AV)	**1.87**	±	**0.05**	0.77***	±	0.06
Anteromedial Nucleus (AM)	**1.71**	±	**0.06**	**1.19****	±	**0.06**
anterior Reticular Thalamus (aRT)	**2.03**	±	**0.03**	**1.37****	±	**0.07**
Globus Pallidus (GP)	0.72	±	0.05	**0.90*****	±	**0.07**
Ventrolateral Nucleus (VL)	0.55	±	0.10	0.31	±	0.08
Habenula (Hab)	**0.99**	±	**0.06**	**1.43***	±	**0.06**
Ventromedial Nucleus (VM)	**1.32**	±	**0.05**	**1.44**	±	**0.06**
Basolateral (BLA)	0.64	±	0.06	0.47	±	0.03
Central (CeA)	**1.22**	±	**0.06**	**0.93**	±	**0.08**
Medial (MeA)	0.64	±	0.04	**1.14***	±	**0.10**
Retrosplenial Cortex (RSC)	**1.09**	±	**0.09**	**1.21**	±	**0.07**
Molecular Layer (DH.Mol)	0.61	±	0.05	0.79	±	0.04
Cornu Ammonis 1 (DH.CA1)	0.69	±	0.08	**1.07**	±	**0.07**
Cornu Ammonis 2 (DH.CA2)	0.81	±	0.06	0.81	±	0.10
Dentate Gyrus (DH.DG)	0.66	±	0.06	**1.00****	±	**0.03**
Medial Geniculate (MG)	0.61	±	0.06	0.81	±	0.04
Molecular Layer (VH.Mol)	0.72	±	0.05	0.80	±	0.06
Dorsal Subiculum (Dsub)	0.79	±	0.07	**1.13**	±	**0.08**
Cornu Ammonis 1 (VH.CA1)	0.82	±	0.08	0.74	±	0.06
Cornu Ammonis 3 (VH.CA3)	**0.88**	±	**0.08**	0.36**	±	0.04
Dentate Gyrus (VH.DG)	0.74	±	0.06	**1.48*****	±	**0.07**
Substantia Nigra pars Reticulata (SNR)	0.47	±	0.05	0.51	±	0.04
Perirhinal Cortex (PRh)	0.73	±	0.11	**0.99****	±	**0.07**
Entorhinal Cortex (EntC)	**0.88**	±	**0.09**	0.37**	±	0.03
Substantia Nigra pars Compacta (SNC)	0.53	±	0.14	**0.84**	±	**0.07**
Mamillary Body (MB)	**0.91**	±	**0.04**	**0.94**	±	**0.08**
Ventral Tegmental Area (VTA)	**1.02**	±	**0.06**	**0.99**	±	**0.08**
Dorsal Raphé (DR)	**1.23**	±	**0.07**	**1.05**	±	**0.08**
Median Raphé (MR)	0.67	±	0.02	**1.42*****	±	**0.08**

Functional connectivity of the mediodorsal thalamic nucleus (MD) and its modification by chronic, intermittent WIN55,212-2 administration. Data shown represent the variable importance to the projection (VIP) as derived from partial least squares regression (PLSR) analysis with the MD as the "seed" brain region (dependent variable) and all other brain regions of interest (RoI) as the explanatory variables. Data were statistically analysed using

Student's t-test with Bonferroni post-hoc correction for multiple comparisons. Bold denote a significant functional connection to the seed region (95% confidence interval of the VIP statistic exceeds 0.8 threshold). *denotes $p<0.05$, **denotes $p<0.01$ and ***denotes $p<0.001$ significant difference from control animals. DH denotes dorsal hippocampus, VH denotes ventral hippocampus.

Table S6. Functional Connectivity of the Globus Pallidus

RoI	Control			WIN55,212-2 Treated		
anterior Prelimbic Cortex (aPrL)	**1.10**	**±**	**0.07**	**0.95**	**±**	**0.06**
Frontal Association Cortex (FRA)	0.71	±	0.10	**1.07**	±	**0.04**
Dorsolateral Orbital Cortex (DLO)	0.72	±	0.05	**1.34****	±	**0.09**
Lateral Orbital Cortex (LO)	**1.01**	**±**	**0.09**	0.70	**±**	0.04
Medial Orbital Cortex (MO)	0.55	±	0.06	0.61	±	0.06
medial Prelimbic Cortex (mPrL)	0.66	±	0.05	**1.30*****	±	**0.05**
Infralimbic Cortex (IL)	0.63	±	0.05	0.71	±	0.07
Nucleus Accumbens Core (AcbC)	**0.90**	**±**	**0.05**	0.53*	**±**	0.05
Nucleus Accumbens Shell (AcbSh)	0.73	±	0.05	0.61	±	0.04
Dorsolateral Striatum (DLST)	**1.61**	**±**	**0.02**	0.52***	**±**	0.03
Ventromedial Striatum (VMST)	**1.02**	**±**	**0.08**	0.57*	**±**	0.05
Medial Septum (MS)	**1.45**	**±**	**0.04**	0.67***	**±**	0.03
Lateral Septum (LS)	0.80	±	0.05	0.73	±	0.05
Ventral Limb Diagonal Band Broca (VDB)	**1.66**	**±**	**0.03**	0.51***	**±**	0.03
orizontal Limb Diagonal Band of Broca (HDB)	**1.39**	**±**	**0.05**	0.52***	**±**	0.10
Piriform Cortex (Piri)	**1.14**	**±**	**0.08**	0.68*	**±**	0.05
Insular Cortex (Ins)	0.54	±	0.04	0.76	±	0.10
Cingulate Cortex (Cg1)	0.59	±	0.04	**1.26*****	±	**0.04**
Somatosensory Cortex (S1)	**1.49**	**±**	**0.07**	0.46***	**±**	0.04
Anteroventral Nucleus (AV)	0.79	±	0.06	**1.03**	±	**0.08**
Anteromedial Nucleus (AM)	**1.01**	**±**	**0.06**	**1.11**	**±**	**0.09**
anterior Reticular Thalamus (aRT)	0.64	±	0.03	0.52	±	0.13
Mediodorsal Nucleus (MD)	0.61	±	0.04	**0.98***	±	**0.07**
Ventrolateral Nucleus (VL)	**1.30**	**±**	**0.03**	**1.14**	**±**	**0.06**
Habenula (Hab)	**1.15**	**±**	**0.03**	**0.93**	**±**	**0.07**
Ventromedial Nucleus (VM)	0.50	±	0.05	**0.97**	±	**0.11**
Basolateral (BLA)	**1.08**	**±**	**0.05**	0.51***	**±**	0.07
Central (CeA)	0.79	±	0.08	0.38*	±	0.03
Medial (MeA)	**1.59**	**±**	**0.04**	**1.09*****	**±**	**0.06**
Retrosplenial Cortex (RSC)	0.38	±	0.07	0.54	±	0.09
Molecular Layer (DH.Mol)	**1.25**	**±**	**0.05**	0.62**	**±**	0.09
Cornu Ammonis 1 (DH.CA1)	**1.17**	**±**	**0.05**	**1.28**	**±**	**0.04**
Cornu Ammonis 2 (DH.CA2)	0.63	±	0.09	**0.98**	±	**0.07**
Dentate Gyrus (DH.DG)	0.45	±	0.10	**0.89**	±	**0.06**
Medial Geniculate (MG)	**1.04**	**±**	**0.05**	**0.97**	**±**	**0.05**
Molecular Layer (VH.Mol)	0.57	±	0.04	0.76	±	0.05
Dorsal Subiculum (VH.DS)	**1.23**	**±**	**0.07**	**2.07*****	**±**	**0.03**
Cornu Ammonis 1 (VH.CA1)	**0.90**	**±**	**0.06**	**0.99**	**±**	**0.06**
Cornu Ammonis 3 (VH.CA3)	**1.55**	**±**	**0.10**	0.79***	**±**	0.03
Dentate Gyrus (VH.DG)	0.50	±	0.05	**1.08***	±	**0.11**
Substantia Nigra pars Reticulata (SNR)	0.50	±	0.03	**0.88*****	±	**0.03**
Perirhinal Cortex (PRh)	0.45	±	0.10	**1.12****	±	**0.05**
Entorhinal Cortex (EntC)	**0.97**	**±**	**0.06**	**1.45**	**±**	**0.09**
Substantia Nigra pars Compacta (SNC)	0.72	±	0.04	0.53	±	0.07
Mamillary Body (MB)	0.50	±	0.09	0.79	±	0.06
Ventral Tegmental Area (VTA)	**1.50**	**±**	**0.04**	**1.10****	**±**	**0.05**
Dorsal Raphé (DR)	0.66	±	0.05	**1.65*****	±	**0.05**
Median Raphé (MR)	0.64	±	0.06	**1.87*****	±	**0.07**

Functional connectivity of the Globus Pallidus (GP) and its modification by chronic, intermittent WIN55,212-2 administration. Data shown represent the variable importance to

the projection (VIP) as derived from partial least squares regression (PLSR) analysis with the GP as the "seed" brain region (dependent variable) and all other brain regions of interest (RoI) as the explanatory variables. Data were statistically analysed using Student's t-test with Bonferroni post-hoc correction for multiple comparisons. Bold denote a significant functional connection to the seed region (95% confidence interval of the VIP statistic exceeds 0.8 threshold). *denotes $p<0.05$, **denotes $p<0.01$ and ***denotes $p<0.001$ significant difference from control animals. DH denotes dorsal hippocampus, VH denotes ventral hippocampus.
